# Corrigendum to: Imaging and cancer: a review

**DOI:** 10.1002/1878-0261.13283

**Published:** 2022-08-03

Leonard Fass [1] would like to state the term ‘short pulses’ should have been used instead of ‘short wavelengths’ when referring to Photoacoustic Imaging throughout the review article. The authors sincerely apologise for their mistake and for any inconvenience caused.
